# Identification of *Staphylococcus aureus* spondylitis in patients with spinal infections using machine learning based on routine laboratory tests: a multicenter retrospective study

**DOI:** 10.1097/JS9.0000000000003035

**Published:** 2025-07-17

**Authors:** Junbao Chen, Kaile Feng, Qianfei Liu, Ping Luo, Tao Li, Chaofeng Guo, Qingfang Zhang, Guang Zhang, Xiheng Hu, Qile Gao

**Affiliations:** a Department of Spine Surgery and Orthopaedics, Xiangya Hospital, Central South University, Changsha, China; b National Clinical Research Center for Geriatric Disorders, Xiangya Hospital, Central South University, Changsha, China; c Department of Pharmacy, Xiangya Hospital, Central South University, Changsha, China; d The Affiliated Changsha Hospital of Xiangya School of Medicine, Central South University, Changsha, China; e Department of Orthopaedics, the First Affiliated Hospital of Shantou University Medical College, Shantou, China; f Furong Laboratory, Changsha, Hunan, China; g National Engineering Research Center of Personalized Diagnostic and Therapeutic Technology, Changsha, Hunan, China

**Keywords:** Logistic Regression (LR), machine learning, Random Forest (RF), Regression (LR), S. aureus spondylitis, SHAP, Spinal infection, Support Vector Machine (SVM), XGBoost

## Abstract

Rapid etiological identification of *Staphylococcus aureus* in spinal infections can be challenging, often delaying targeted therapy. We developed a machine learning model leveraging XGBoost to predict *S. aureus* etiology in spinal infections directly from routine laboratory indicators. The XGBoost model demonstrated superior predictive performance (AUC 0.812; 95% CI: 0.728-0.896) among four algorithms, with SHAP analysis identifying D-dimer, Monocyte Percentage, Albumin, and Alanine Aminotransferase as crucial predictors.

In accordance with the Transparency In The Reporting of Artificial Intelligence (TITAN) guideline^[1]^, which establishes standards for AI-related medical research reporting, we explicitly state that no generative AI tools were used in this study design, data analysis, or manuscript preparation. The reporting of this diagnostic accuracy study follows the Standards for Reporting Diagnostic Accuracy Studies (STARD) 2015 guidelines. The completed STARD checklist is provided as Supplementary Digital Content Material, available at: http://links.lww.com/JS9/E693.

**HIGHLIGHTS**

- Machine learning models differentiate *S. aureus* from non-*S. aureus* spinal infections using routine laboratory tests.
- The XGBoost algorithm demonstrated superior diagnostic performance for *Staphylococcus aureus* spondylitis among comparative machine learning models, achieving the highest discriminative capability with an AUC of 0.812.
- D-dimer (DD), Monocyte Percentage (Mono%), Albumin (A), and alanine aminotransferase (AST) are top-ranked predictors for distinguishing *S. aureus*.

*S. aureus* spondylitis is a severe spinal infection, potentially life-threatening, demanding prompt and accurate diagnosis for effective treatment^[2]^. Differentiating *S. aureus* from other pathogens is crucial for tailoring antimicrobial therapy, yet conventional methods like cultures and imaging are often slow, invasive, or lack sufficient specificity^[3]^. To address this, we developed machine learning (ML) models leveraging routine laboratory indicators for rapid identification.

Machine learning, utilizing computational analytics on routinely collected data, offers a promising approach for rapid, non-invasive diagnostics^[4,5]^. Given that *S. aureus* infections often exhibit accelerated progression and greater clinical severity compared to those caused by other pathogens, our study employs ML with readily accessible laboratory parameters to predict *S. aureus* etiology, aiming to facilitate timely and appropriate therapeutic interventions.

We enrolled 487 patients with microbiologically confirmed spinal infections from five hospitals. Patients were categorized into *S. aureus* spondylitis (n = 110) and non-*S. aureus* spondylitis (n = 377) groups based on mNGS and culture. The dataset was randomly partitioned into a training cohort (n = 341) and a test cohort (n = 146) (Fig. 1). Routine laboratory indicators were selected as input features following Boruta algorithm-based feature selection. While univariate analysis identified significant differences (p < 0.001, Supplementary Digital Content Table 1, available at: http://links.lww.com/JS9/E691) in specific variables between *S. aureus* and non-*S. aureus* spondylitis cohorts, individual biomarkers demonstrated limited diagnostic efficacy (all ROC AUCs < 0.8) (Fig. 1A, B). To address multicollinearity and mitigate overfitting, the Boruta algorithm identified 20 significant biomarkers (including hematological parameters (WBC, RBC, PLT, Neut, BASO, BASO%, PCT, Neut%, Mono%), inflammatory and coagulation markers (C-reactive protein, erythrocyte sedimentation rate, DD, activated partial thromboplastin time), and biochemical indicators (total protein, A, A/G, cholesterol, AST, high-density lipoprotein, creatinine) spanning hematological, inflammatory/coagulation, and biochemical profiles for predicting *S. aureus* spondylitis (Fig. 1C, D).

**Figure 1.**
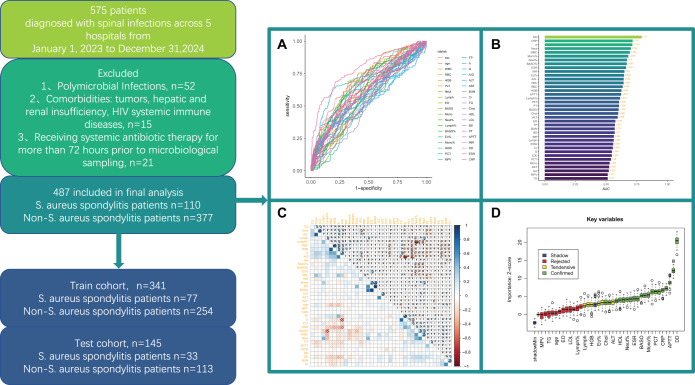
Flow diagram of study design. A: ROC curves for 38 routine laboratory test results; B: AUC for 38 routine laboratory test results; C: The heat map of correlations between continuous variables demonstrates the existence of covariance between many variables; D: Boruta’s algorithm (based on random forests).

We developed models using LR, RF, SVM, and XGBoost. Model performance was evaluated on a 30% test set based on the AUC. The AUCs and confusion matrix of the different models were as follows (Fig. 2, Supplementary Digital Content Table 2, available at: http://links.lww.com/JS9/E692): LR, 0.719(0.613,0.824); RF, 0.803(0.718,0.889); SVM, 0.769(0.681,0.856); XGBoost, 0.812(0.728,0.896); Comparison of the AUC values revealed that XGBoost demonstrated superior predictive performance relative to the other models. Calibration curve and clinical decision curve of XGBoost were shown in Fig. 2.

**Figure 2.**
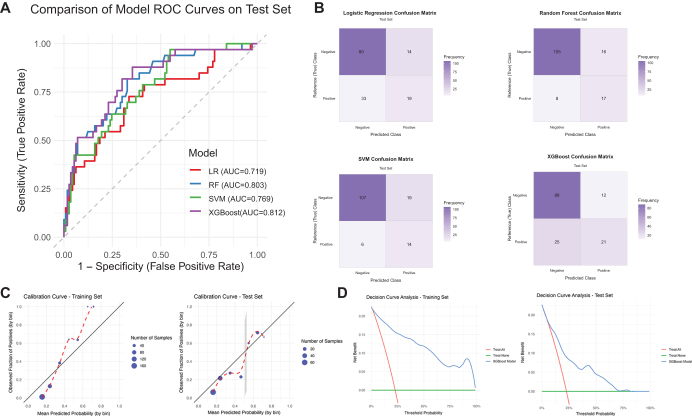
Model performance. A: ROC of models in test set. B: Confusion matrix of models in test set. C: XGBoost model performance evaluation. Calibration curve of the model on the training set and test set. D. Clinical decision curve of the model on the training set and test set.

To interpret the XGBoost model, SHAP analysis ranked predictor importance, identifying D-dimer (DD), Albumin (A), AST, and Monocyte Percentage (Mono%) as the most influential (Fig. 3A). SHAP values also elucidated relationships, such as higher DD positively correlating with *S. aureus* spondylitis while higher albumin showed a negative correlation (Fig. 3B). Individual patient force plots (Fig. 3C, 3D) visualized feature contributions to specific predictions. Furthermore, SHAP dependence plots (Fig. 3E, F, G, H) detailed how these Top 4 features influenced model output across their value ranges, with SHAP values > 0 indicating a positive contribution towards predicting *S. aureus*, while marginal density distributions provided insight into their spread.

**Figure 3.**
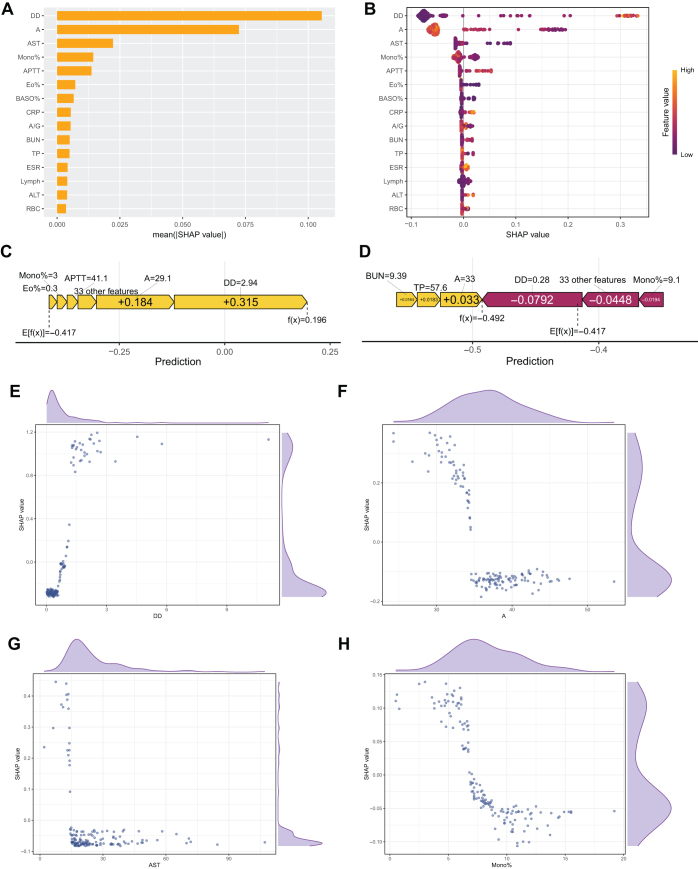
A: The weights of variables importance; B: The SHAP values of variables importance; C: SHAP Individual Force Plots for *S. aureus* spondylitis patient; D: SHAP Individual Force Plots for *Non-S. aureus* spondylitis patient. E, F, G, H: The univariate dependence plots and marginal density distribution of values and SHAP values for the Top 4 variables (DD, A, AST, Mono%) of importance in test set.

This study built and validated four machine learning models—LR, RF, SVM, and XGBoost—for identifying *S. aureus* spondylitis from routine laboratory indicators, forming a potentially rapid diagnostic pipeline. Validated on an independent test cohort, the XGBoost model demonstrated superior performance. The application of SHAP analysis enhanced model interpretability, highlighting DD, Mono%, A, and AST as the most influential predictors.

The top SHAP-identified predictors have strong pathophysiological relevance to *S. aureus* infections. Elevated DD reflects *S. aureus*’s known procoagulant effects (coagulase, alpha toxin) and associated hypercoagulability^[6,7]^. Increased Mono% indicates systemic inflammatory burden and immune activation, consistent with monocyte recruitment and response to staphylococcal pathogens^[8]^. Reduced A levels often signify systemic inflammation, malnutrition, and increased vascular permeability common in severe infections, while elevated AST can indicate hepatic involvement secondary to *S. aureus* infection. Previous studies indicate that *S. aureus* infection can impair liver function and cause hepatic injury by interfering with normal metabolic processes^[9]^.

This XGBoost model, utilizing key predictors from routine lab tests, enhances clinical decision-making for spinal infections by providing an early, data-driven probability of *S. aureus* etiology, significantly faster than microbiological confirmation. This facilitates timely, targeted empiric antibiotic therapy and diagnostic planning, effectively shortening the diagnostic timeline, especially if integrated into EHR-based tools.

The multicenter design and use of accessible serological biomarkers are strengths enabling rapid prediction. However, a key limitation is the exclusion of clinical and radiographic parameters, known to aid diagnosis in prior studies^[10]^. Future work will focus on integrating these data.

Despite limitations, the model demonstrates excellent predictive ability for *S. aureus* spondylitis using routine labs. It offers a pragmatic tool to reduce diagnostic delays, enabling prompt intervention and improved antibiotic stewardship, particularly in resource-limited settings. Future research requires multicenter external validation and mechanistic studies of identified biomarkers.

## Data Availability

The authors will provide the raw data supporting the findings of this article upon request, without any unwarranted delays or restrictions. For additional questions or inquiries, please contact the corresponding author.

## Research registration unique identifying number (UIN)

UIN: ChiCTR2400081336.
